# Application of artificial intelligence in a real-world research for predicting the risk of liver metastasis in T1 colorectal cancer

**DOI:** 10.1186/s12935-021-02424-7

**Published:** 2022-01-15

**Authors:** Tenghui Han, Jun Zhu, Xiaoping Chen, Rujie Chen, Yu Jiang, Shuai Wang, Dong Xu, Gang Shen, Jianyong Zheng, Chunsheng Xu

**Affiliations:** 1 Xijing Hospital, Airforce Medical University, Xi’an, China; 2 State Key Laboratory of Cancer Biology, Institute of Digestive Diseases, Xijing Hospital, Airforce Medical University, Xi’an, China; 3 Department of General Surgery, The Southern Theater Air Force Hospital, Guangzhou, China; 4 Ming Gang Station Hospital, Xi’an Institute of Flight of the Air Force, Minggang, China; 5 School of Clinical Medicine, Xi’an Medical University, Xi’an, China; 6 Division of Digestive Surgery, Xijing Hospital of Digestive Diseases, Airforce Medical University, Xi’an, China

**Keywords:** Artificial intelligence, Machine learning, T1 colorectal cancer, Real-world research, Liver metastasis

## Abstract

**Background:**

Liver is the most common metastatic site of colorectal cancer (CRC) and liver metastasis (LM) determines subsequent treatment as well as prognosis of patients, especially in T1 patients. T1 CRC patients with LM are recommended to adopt surgery and systematic treatments rather than endoscopic therapy alone. Nevertheless, there is still no effective model to predict the risk of LM in T1 CRC patients. Hence, we aim to construct an accurate predictive model and an easy-to-use tool clinically.

**Methods:**

We integrated two independent CRC cohorts from Surveillance Epidemiology and End Results database (SEER, training dataset) and Xijing hospital (testing dataset). Artificial intelligence (AI) and machine learning (ML) methods were adopted to establish the predictive model.

**Results:**

A total of 16,785 and 326 T1 CRC patients from SEER database and Xijing hospital were incorporated respectively into the study. Every single ML model demonstrated great predictive capability, with an area under the curve (AUC) close to 0.95 and a stacking bagging model displaying the best performance (AUC = 0.9631). Expectedly, the stacking model exhibited a favorable discriminative ability and precisely screened out all eight LM cases from 326 T1 patients in the outer validation cohort. In the subgroup analysis, the stacking model also demonstrated a splendid predictive ability for patients with tumor size ranging from one to50mm (AUC = 0.956).

**Conclusion:**

We successfully established an innovative and convenient AI model for predicting LM in T1 CRC patients, which was further verified in the external dataset. Ultimately, we designed a novel and easy-to-use decision tree, which only incorporated four fundamental parameters and could be successfully applied in clinical practice.

**Supplementary Information:**

The online version contains supplementary material available at 10.1186/s12935-021-02424-7.

## Introduction

Colorectal cancer (CRC) is universally acknowledged as one of the most prevalent gastrointestinal tract malignancies with considerably high morbidity and mortality, drawing more and more attention annually [1–3]. In 2/3 of CRC patients, metastasis is commonly recognized as both a pivotal clinical feature and a risk factor of high mortality for intractable CRC [4]. During the progression of CRC, over 50% of patients tend to develop liver metastasis (LM) which is the predominant contributor to unfavorable prognosis of CRC [4, 5]. Synchronous LM is determined at the time of diagnosis and 15–25% CRC patients had synchronous LM [6, 7].

Endoscopic therapy is a widely accepted and adopted as a valid therapeutic method for T1 CRC patients. Nonetheless, for early CRC patients with LM, conventional surgical excision, neoadjuvant chemotherapy and radiofrequency ablation are the most effective and recommended treatments, which significantly prolong the 5-year overall survival (OS) rate of CRC patients [8, 9]. However, considering the inferior early screening methods, approximately 90% of CRC patients with LM fail to be diagnosed precisely in the early stage and thus undergo incomplete endoscopic resection, which ultimately gives rise to undesirable clinical outcomes [10, 11]. Although scholars and academicians have conducted abundant in-depth researches on metastasis-related signatures in vivo and vitro, a satisfactory predictive model of LM for CRC in the early stage is still lacking [12–14]. Consequently, we aimed at developing an easy-to-use model to predict the risk of LM for patients in the early stage of CRC accurately and robustly.

Currently, there exists an upregulating and irreversible tendency of discipline integration between medical science and artificial intelligence (AI) [15–17]. Besides, both depth and breadth of the discipline integration have been significantly enhanced [14, 15]. Researchers employed machine learning (ML) as the breaking point in solving the complicated issue of CRC clinical prediction and acquired plentiful significant breakthroughs [18–20]. Nevertheless, these findings simply shed light on the intriguing area of T1 CRC with lymph node metastasis which resembles a virgin land to be further explored by utilizing ML. Given that the majority of previous investigations merely concentrated on the public database when studying the apparent discrepancy among diverse populations, limitations ineluctably appeared. Consequently, clinical data involving the real outer validation is of vital significance to construct a superior prediction model.

In the study, we developed a comprehensive recognition model via adopting AI and ML algorithms, which could remarkably promote the identification of T1 CRC with LM and improve the prognosis of these patients in clinical practice. In addition, the predictive model was constructed via utilizing clinically common and accessible parameters, and further validated in an independent CRC cohort.

## Materials and methods

### Clinical sample collection

An open-access and publicly available CRC cohort was retrieved from Surveillance, Epidemiology, and End Results (SEER) Program database in the U.S. National Cancer Institute. The CRC cohort functioned as a powerful resource for investigators to comprehensively comprehend the natural history of CRC and significantly ameliorated the healthcare quality for CRC patients [21, 22]. An additional outer validation cohort of CRC patients who underwent surgery from 2010 to 2021 was obtained from Xijing hospital. The CRC cohort's inclusive criteria were demonstrated as follows: (1) the primary diagnosis was CRC; (2) patients were diagnosed with T1 CRC; (3) liver reexamination was completed within six months of diagnosis; (4) patients with sufficient clinical data. Additionally, exclusive criteria were exhibited as follows: (1) patients who have undergone neoadjuvant radiotherapy; (2) metachronous liver metastases (after diagnosis); (3) comorbidity with other tumors; (4) comorbidity with serious cardiopulmonary disease. Written and informed consent was obtained from all participants. All aspects of the clinical cohort study were evaluated by and included in the Institutional Ethics Committee of Xijing Hospital.

### Study population

T1 CRC is defined as a category of tumor that invades only the submucosa, regardless of the presence or absence of lymph node metastasis (LNM). Utilizing the SEER database which employed the 7th cancer TNM stages of the American Joint Committee, we analyzed the data of all patients diagnosed with T1 CRC from 2010 to 2016. Primary demographic data, tumor information and laboratory indexes were extracted by utilizing SEER disease codes and then employed for model construction. Fundamental demographic data included age at diagnosis, gender, race, and marital status. Tumor information contained primary site, size, grade, histologic category and TNM stage. Laboratory indexes involved carcinoembryonic antigen (CEA) prior to surgery, tumor deposits, and perineural invasion (PNI). Survival time and status were collected for further clinical estimation of the predictive model. Furthermore, the information of our validation cohort was normalized via following the criteria of the SEER database (Additional file 1: Table S1). And all clinical information underwent data transformation for the sake of further application in model construction (Additional file 2: Table S2).

### Construction of the predictive model

In our research, seven ML models were employed to predict LM in patients with T1 stage CRC. To build up tree decision models, we adopted Light Gradient Boosting Decision (LGBM), Random Forest (RF), and Classification and Regression Trees (CART). LGBM is a gradient boosting framework that utilizes the tree-based learning algorithm, which has been successfully applied in the construction of medical models in recent years [23, 24]. RF is a universally employed ML algorithm to deal with classification and regression issues via the multiple decision trees approach [25]. CART is a classical decision tree algorithm applied in either classification or regression predictive models [26]. The K-Nearest Neighbor (KNN) algorithm was utilized in basic prediction technique. KNN is identified as a vital classification algorithm in the supervised ML domain and is extensively applied in pattern recognition, data mining and intrusion detection [27]. To construct the kernel-based model, the Support Vector Machine (SVM) was selected and put into use. SVM is a supervised ML model that employs classification algorithms for the two-group categorization [28]. Gaussian Naive Bayesian (GNB) algorithm was included in the linear model for specific utilization under the circumstance where the features manifested continuous values [29]. Multilayer Perceptron (MLP) is a feed-forward neural network supplement and has been extensively applied in distinct prediction models [30]. In the wake of employing the Bootstrap aggregating (Bagging) algorithm to optimize the performance of established models, stacked regression was utilized to obtain a stacking model via integrating seven models to output a desirable outcome [31, 32].

**Fig. 1:**
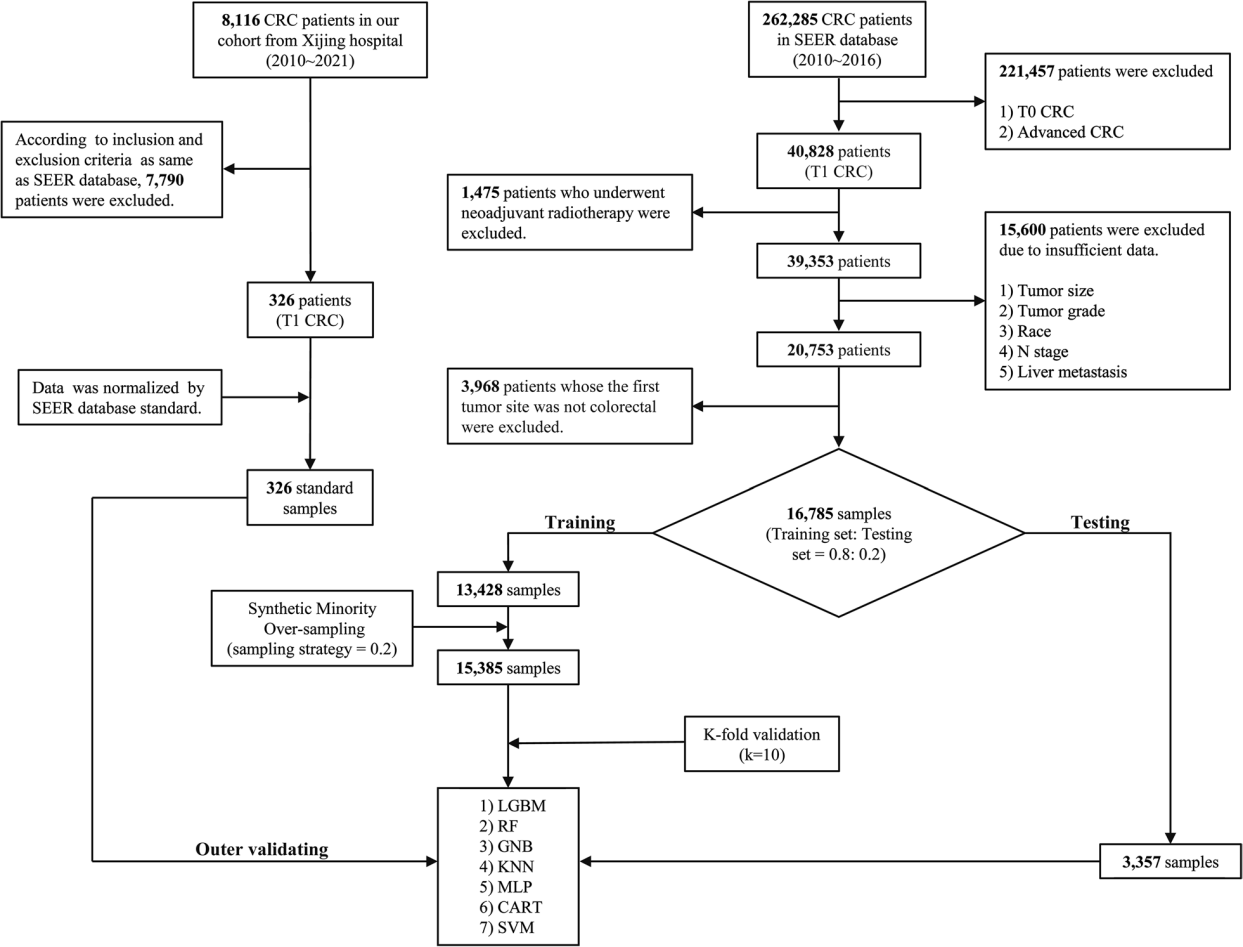
The workflow of selection procedure for colorectal cancer patients

To polish up performance of the model and retain maximum authenticity of the data, we strictly employed the Synthetic Minority Over-sampling technique in the inner training dataset to solve the issue of data imbalance [33]. To begin with, patients in the SEER database were randomly assigned to the training set (80%) and testing set (20%) respectively while the proportion of LM ( +) (patients with LM) subgroup was approximately identical to that of the LM (−) (patients without LM) subgroup (Additional files 12 and 13). In the training set, k-fold cross validation (k = 10) was performed, and grid search was adopted to figure out the best combination of parameters. For each set of parameters, the model was in turn fitted and validated with 8/10 and 2/10 of data respectively. Subsequently, our T1 CRC cohort in the Chinese population was utilized as an extra outer validation set further to examine both applicability and efficiency of the model (Additional file 14). The overall workflow is elaborately demonstrated in Fig. 1.

### Assessment of model performance

To ensure rational comparison of the models and assess their performance, a multitude of indicators were employed involving confusion matrix, the area under the curve (AUC), sensitivity, specificity, precision, negative predictive value (NPV), false discovery rate (FDR), accuracy, and average precision (AP). In addition, the area under receiver operating characteristic curves (AU-ROC) was utilized as a performance index while the AP value was employed as the criterion for the precision-recall (PR) curve [34]. The average value of parameters was ultimately executed on the testing set and additional outer validation one. Survival analysis was further adopted in the model to evaluate whether it was capable of accurately predicting CRC patients’ outcomes.

In light of the fact that neoplastic size was widely recognized as an effective predictor of CRC outcome, we tested nonlinearity of the model via analysis of 5-knot restricted cubic splines (RCS) and evaluated potential correlation of model with the hazard of LM [35]. In order to estimate the performance of models in patients with small CRC sizes, we stratified the testing set into 4 subgroups, tumor sizes of which being 1–10 mm, 1–20 mm, 1–50 mm and > 50 mm respectively. Their AUC and AP values were then calculated.

Moreover, to make the real clinical decision process more reliable, training samples were adopted prior to utilization of over-sampling strategy. Subsequently, to exhibit the specific decision process of how CRC patients with LM were discriminated from the model, regression tree analysis was conducted via CART algorithm.

### Statistical analysis

SEER*Stat software (8.3.6 version) was adopted to acquire targeted CRC patients from the SEER database. Python (version 3.6.9) and R software (version 4.0.5) were utilized to perform statistical analyzes. Python packages were listed: ‘imblearn’, ‘sklearn’, ‘lightgbm’, and ‘mlxtend’. R packages were vividly demonstrated as follows: ‘tableone’, ‘survival’, ‘mice’, and ‘dplyr’. Demographic differences between the two subgroups were tested utilizing either Student’s t-test or Pearson chi‐square test. Results were considered statistically significant when P ≤ 0.05.

## Results

### Case structures and clinical baselines

Included CRC data in our study from SEER database ranged from 2010 to 2016. In the aggregate, 262,285 CRC patients were initially enrolled. According to the inclusive and exclusive criteria, a totality of 16,785 patients were enrolled in the inner dataset and 326 out of 8226 CRC patients in Xijing hospital were recruited ultimately (Fig. 1). Baseline clinical characteristics of the SEER CRC cohort (Training dataset) and Xijing CRC cohort (Validation dataset) were exhibited in detail (Table 1).

**Table 1:** Clinical baseline features of SEER and Xijing hospital database

Variables	SEER database		Xijing CRC cohort
	Training set	Testing set	Outer validation set
Age at diagnosis, n (%)			
0–9	14 (0.1)	0(0)	0(0)
10–19	128 (1.0)	31 (0.9)	0(0)
20–29	265 (2.0)	67 (2.0)	4 (1.2)
30–39	390 (2.9)	79 (2.4)	5 (1.5)
40–49	1084 (8.1)	251 (7.5)	35 (10.7)
50–59	3632 (27.0)	945 (28.2)	104 (31.9)
60–69	3649 (27.2)	911 (27.1)	92 (28.2)
70–79	2659 (19.8)	670 (20.0)	65 (19.9)
80–89	1403 (10.4)	354 (10.5)	21 (6.4)
90–99	204 (1.5)	49 (1.5)	0(0)
Gender, n (%)			
Female	6982 (52.0)	1695 (50.5)	189 (58.0)
Male	6446 (48.0)	1662 (49.5)	137 (42.0)
Race, n (%)			
White	10,226 (76.2)	2552 (76.0)	0(0)
Black	1754 (13.1)	466 (13.9)	0(0)
Asian or Pacific Islander	1354 (10.1)	319 (9.5)	326 (100.0)
American Indian/Alaska Native	94 (0.7)	20 (0.6)	0(0)
Marital status at diagnosis, n (%)			
Married and separated	7615 (56.7)	1855(55.2)	322 (98.8)
Divorced	1207 (9.0)	293 (8.7)	2 (0.6)
Unmarried	2219 (16.5)	559 (16.7)	2 (0.6)
Other	2387(17.8)	650(19.3)	0(0)
LM, n (%)			
Yes	12,821 (95.5)	3202 (95.4)	318 (97.5)
No	607 (4.5)	155 (4.6)	8 (2.5)
Primary site, n (%)			
Rectum, NOS	3786 (28.2)	955 (28.4)	228 (69.9)
Sigmoid colon	2925 (21.8)	777 (23.1)	35 (10.7)
Ascending colon	1646 (12.3)	413 (12.3)	24 (7.4)
Cecum	1586 (11.8)	393 (11.7)	6 (1.8)
Appendix	868 (6.5)	216 (6.4)	0(0)
Rectosigmoid junction	846 (6.3)	215 (6.4)	7 (2.1)
Transverse colon	723 (5.4)	166 (4.9)	9 (2.8)
Descending colon	481 (3.6)	106 (3.2)	1 (0.3)
Hepatic flexure of colon	303 (2.3)	70 (2.1)	7 (2.1)
Splenic flexure of colon	172 (1.3)	31 (0.9)	3 (0.9)
Colon, NOS	50 (0.4)	9 (0.3)	4 (1.2)
Overlapping lesion of colon	42 (0.3)	6 (0.2)	2(0.6)
Tumor size, mm, mean (SD)	19.16 (25.1)	18.82 (22.3)	24.6 (14.0)
Tumor grade, n (%)			
Well differentiated; Grade I	4171 (31.1)	1015 (30.2)	69 (21.2)
Moderately differentiated; Grade II	8306 (61.9)	2114 (63.0)	240 (73.6)
Poorly differentiated; Grade III	827 (6.2)	191 (5.7)	15 (4.6)
Undifferentiated; anaplastic; Grade IV	124 (0.9)	37 (1.1)	2 (0.6)
Tumor type, n (%)			
Adenocarcinoma, NOS	4368 (32.5)	1099 (32.7)	91 (27.9)
Adenocarcinoma in tubulovillous adenoma	2969 (22.1)	743 (22.1)	76 (23.3)
Adenocarcinoma in adenomatous polyp	2827 (21.1)	708 (21.1)	125 (38.3)
Carcinoid tumor, NOS	1837 (13.7)	454 (13.5)	0(0)
Adenocarcinoma in villous adenoma	483 (3.6)	126 (3.8)	9 (2.8)
Neuroendocrine carcinoma, NOS	409 (3.0)	93 (2.8)	0(0)
Mucinous adenocarcinoma	238 (1.8)	61 (1.8)	7 (2.1)
Squamous cell carcinoma, NOS	52 (0.4)	8 (0.2)	0(0)
Atypical carcinoid tumor	38 (0.3)	11 (0.3)	0(0)
Signet ring cell carcinoma	28 (0.2)	6 (0.2)	0(0)
Mucin-producing adenocarcinoma	26 (0.2)	6 (0.2)	0(0)
Tubular adenocarcinoma	22 (0.2)	8 (0.2)	18 (5.5)
Gastrointestinal stromal sarcoma	17 (0.1)	0(0)	0(0)
Carcinoma, NOS	14 (0.1)	5 (0.1)	0(0)
Villous adenocarcinoma	10 (0.1)	2 (0.1)	0(0)
Other	90 (0.7)	27 (0.8)	0(0)
N, n (%)			
N0	12,142 (90.4)	3031 (90.3)	295 (90.5)
N1	1150 (8.6)	296 (8.82)	30 (9.2)
N2	136 (1.0)	30 (0.9)	1 (0.3)
CEA, n (%)			
Positive	1223 (9.1)	300 (8.9)	110 (33.7)
Borderline	25 (0.2)	6 (0.2)	0(0)
Negative	3974 (29.6)	993 (29.6)	200 (61.3)
Unknown	8206 (61.1)	2058 (61.3)	16 (4.9)
Tumor deposits, n (%)			
No tumor deposits	8777 (65.4)	2213 (65.9)	325 (99.7)
Tumor Deposits identified	95 (0.7)	27 (0.8)	1 (0.3)
Unknown	4556 (33.9)	1117 (33.3)	0(0)
Perineural invasion, n (%)			
Yes	9104 (67.8)	2246 (66.9)	169 (51.8)
No	105 (0.8)	48 (1.4)	157 (48.2)
Unknown	4219 (31.4)	1063 (31.7)	0(0)

*SEER* Surveillance, Epidemiology, and End Results, *CRC* colorectal cancer, *LM* liver metastasis, *NOS* not otherwise specified, *SD* standard deviation, *CEA* carcinoembryonic antigen

Eleven independent clinical factors were included in our established model, incorporating age at diagnosis, gender, marital status at diagnosis, primary site, tumor size, tumor grade, tumor type, N stage, CEA level, tumor deposits, and PNI. Patients from the SEER database were categorized into LM (−) subgroup (16,023 patients without LM, 95.5%) and LM (+) (762 patients with LM, 4.5%) subgroup respectively. For diagnosed age, we found that the proportion of patients under 60 years of age in LM (+) subgroup (333/762; 43.7%) significantly surpassed that in LM (−) subgroup (6553/16,023; 40.9%; P < 0.001). Notably, the ratio of male CRC was significantly higher in LM (+) subgroup than in its counterpart (P = 0.001). Intriguing, there demonstrated no statistical difference in terms of race between the two subgroups. In line with our anticipation, an upregulated occurrence rate was observed in the single (167/2611, 6.4%) than the married (376/8918, 4.2%; P < 0.001). Regarding tumor sites, rectum was the most common primary site in both subgroups, and the proportion is comparatively higher than other T stages CRC patients (P < 0.001). In respect to progression of CRC, the average tumor size of LM (+) subgroup (52.1 mm) was considerably larger than that of LM (−) one (17.5 mm; P < 0.001). Analogously, LM (+) subgroup demonstrated significantly higher proportions of both Grade II-IV (92.8% vs 68%; P < 0.001) and advanced N stage CRC than LM (−) subgroup (P < 0.001). Furthermore, we observed upregulated levels of tumor deposits, PNI and positive rate of CEA in LM (+) subgroup than its counterpart (P < 0.001). As for tumor differentiation, Adenocarcinoma (Adenocarcinoma, NOS, Adenocarcinoma in tubulovillous adenoma and Adenocarcinoma in adenomatous polyp; 12714/16785, 75.7%) was confirmed as the most common neoplastic category among T1 patients (Table 2).

**Table 2:** Distributions of clinicopathological characteristics in two groups

Variables	LM (−)	LM (+)	P value
	N = 16,023	N = 762	
Age at diagnosis, n (%)			< 0.001
0–9	14 (0.1)	0 (0.0)	
10–19	158 (1.0)	1 (0.1)	
20–29	324 (2.0)	8 (1.0)	
30–39	447 (2.8)	22 (2.9)	
40–49	1238 (7.7)	97 (12.7)	
50–59	4372 (27.3)	205 (26.9)	
60–69	4363 (27.2)	197 (25.9)	
70–79	3185 (19.9)	144 (18.9)	
80–89	1679 (10.5)	78 (10.2)	
90–99	243 (1.5)	10 (1.3)	
Gender, n (%)			
Female	7784 (48.6)	324 (42.5)	0.001
Male	8239 (51.4)	438 (57.5)	
Race, n (%)			0.215
White	12,213 (76.2)	565 (74.1)	
Black	2100 (13.1)	120 (15.7)	
Asian or Pacific Islander	1601 (10.0)	72 (9.4)	
American Indian/Alaska Native	109 (0.7)	5 (0.7)	
Marital status at diagnosis, n (%)			< 0.001
Married	8918 (55.7)	376 (49.3)	
Single	2611 (16.3)	167 (21.9)	
Widowed	1740 (10.9)	90 (11.8)	
Divorced	1417 (8.8)	83 (10.9)	
Unknown	1131 (7.1)	36 (4.7)	
Separated	166 (1.0)	10 (1.3)	
Unmarried or Domestic Partner	40 (0.2)	0 (0.0)	
Primary site, n (%)			< 0.001
Rectum, NOS	4502 (28.1)	239 (31.4)	
Sigmoid colon	3540 (22.1)	162 (21.3)	
Ascending colon	1969 (12.3)	90 (11.8)	
Cecum	1884 (11.8)	95 (12.5)	
Appendix	1081 (6.7)	3 (0.4)	
Rectosigmoid junction	967 (6.0)	94 (12.3)	
Transverse colon	863 (5.4)	26 (3.4)	
Descending colon	569 (3.6)	18 (2.4)	
Hepatic flexure of colon	356 (2.2)	17 (2.2)	
Splenic flexure of colon	194 (1.2)	9 (1.2)	
Colon, NOS	53 (0.3)	6 (0.8)	
Overlapping lesion of colon	45 (0.3)	3 (0.4)	
Tumor size, mm, mean (SD)	17.5 (22.5)	52.1 (39.2)	< 0.001
Tumor grade, n (%)			< 0.001
Well differentiated; Grade I	5131 (32.0)	55 (7.2)	
Moderately differentiated; Grade II	9853 (61.5)	567 (74.4)	
Poorly differentiated; Grade III	891 (5.6)	127 (16.7)	
Undifferentiated; anaplastic; Grade IV	148 (0.9)	13 (1.7)	
Tumor type, n (%)			< 0.001
Adenocarcinoma, NOS	4859 (30.3)	608 (79.8)	
Adenocarcinoma in tubulovillous adenoma	3669 (22.9)	43 (5.6)	
Adenocarcinoma in adenomatous polyp	3495 (21.8)	40 (5.2)	
Carcinoid tumor, NOS	2287 (14.3)	4 (0.5)	
Adenocarcinoma in villous adenoma	596 (3.7)	13 (1.7)	
Neuroendocrine carcinoma, NOS	495 (3.1)	7 (0.9)	
Mucinous adenocarcinoma	281 (1.8)	18 (2.4)	
Squamous cell carcinoma, NOS	59 (0.4)	1 (0.1)	
Atypical carcinoid tumor	49 (0.3)	0 (0.0)	
Signet ring cell carcinoma	32 (0.2)	2 (0.3)	
Mucin-producing adenocarcinoma	30 (0.2)	2 (0.3)	
Tubular adenocarcinoma	30 (0.2)	0 (0.0)	
Gastrointestinal stromal sarcoma	17 (0.1)	0 (0.0)	
Villous adenocarcinoma	12 (0.1)	0 (0.0)	
Carcinoma, NOS	11 (0.1)	8 (1.0)	
Other	101 (0.6)	16 (2.1)	
N, n (%)			< 0.001
N0	14,711 (91.8)	462 (60.6)	
N1	1179 (7.4)	267 (35.0)	
N2	133 (0.8)	33 (4.3)	
CEA, n (%)			< 0.001
Positive	999 (6.2)	524 (68.8)	
Negative	4899 (30.6)	68 (8.9)	
Borderline	28 (0.2)	3 (0.4)	
Unknown	10,097 (63.0)	167 (21.9)	
Tumor deposits, n (%)			< 0.001
No tumor deposits	10,867 (67.8)	123 (16.1)	
Tumor Deposits identified	111 (0.7)	11 (1.4)	
Unknown	5045 (31.5)	628 (82.4)	
Perineural invasion, n (%)			< 0.001
No	11,040 (68.9)	310 (40.7)	
Yes	143 (0.9)	10 (1.3)	
Unknown	4840 (30.2)	442 (58.0)	

*LM* liver metastasis, *NOS* not otherwise specified, *SD* standard deviation, *CEA* carcinoembryonic antigen

### Parameters tuning in our models

We trained the LGBM with a depth of five, a learning rate of 0.01, basic learners of 240, leaves of 16, and max bins of 128. For RF and CART, we also elected 5 as the maximum depth of the basic trees. The number of neighbors 200 for KNN was the best. In MLP, we ultimately selected the learning rate of 0.01, epochs of 300, hidden layer of 1, and utilized the Adam Optimizer and ReLU activation function. For SVM, a combination of a C value of 0.01 and kernel smoothing parameters of 0.0001 was determined as the ultimate choice. Additionally, every Bagging model, in possession of 10 basic models, was trained with identical algorithms but various data. The ultimate stacking model incorporated seven bagging models, probability and GNB output by which were recognized as meta classifier.

### Evaluation of models

**Fig. 2:**
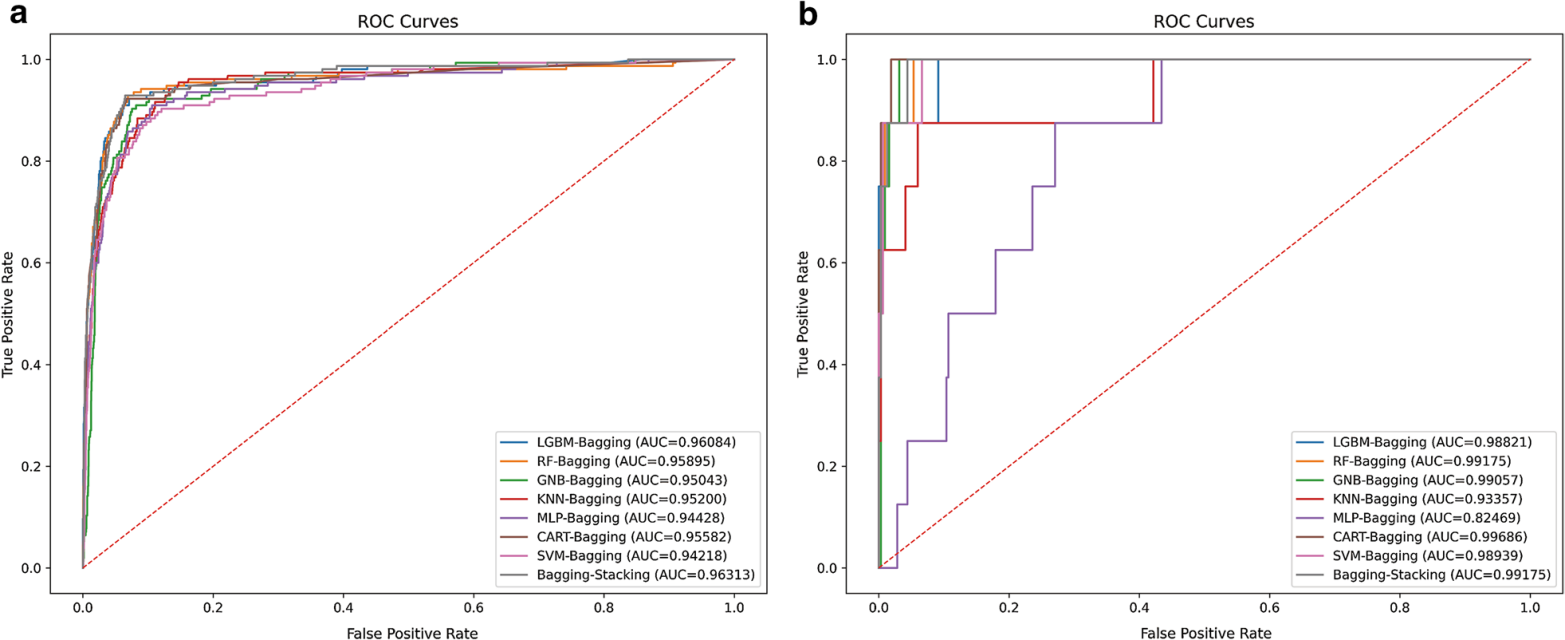
Predictive value of overall models after optimization. Inner validation in SEER database: **a** ROC curves of seven individual models and stacking model. Outer validation in our Chinese cohort: **b** ROC curves of seven individual models and stacking model. SEER: Surveillance, Epidemiology, and End Results; and ROC: receiver operating characteristic

Via internal verifying, all models were observed to reveal superior predictive abilities (AUC values > 0.94). Moreover, by incorporating seven other single models, the stacking model demonstrated a favorable AUC of up to 0.9631 (Fig. 2a). Except for GNB models, AP values of approximately all models attain comparatively preferable levels. Noticeably, the ultimate AP of the stacking mode reached 0.693 (Additional file 3: Figure S1a). Expectedly, the external validation set demonstrated satisfying performance. All models exhibited dramatically high predictive value except the MLP model, and the stacking model contained a final AUC value of 0.992 and an ultimate AP value of 0.811 (Fig. 2b and Additional file 3: Fig. S1b). Additionally, via employing the confusion matrix to appraise the value of models, predictive outcomes of both the inner testing set and outer validation set were displayed in Table 3. LGBM produced fewer quantities of FN (False Negative) and FP (False Positive) than other models in both testing sets. The stacking model was capable of screening approximately all LM (+) patients in both sets. Detailed values of AUC, sensitivity, specificity, precision, NPV, FDR, accuracy, AP, F1-values, and Matthews correlation coefficient of each model in inner and outer validation sets were listed respectively in Additional file 4: Table S3 and Additional file 5: Table S4. The accuracy of five single models reached 0.95, among which LGBM displayed the highest precision (0.9657). The specificity of MLP and sensitivity of GNB were the highest among seven single models. Taken together, the stacking model consistently outperformed other single ML models.

**Table 3:** Confusion matrices of developed models

Confusion matrix	Inner validation			Outer validation		
	Actual	Prediction		Actual	Prediction	
		LM (−)	LM (+)		LM (−)	LM (+)
LGBM	LM (+)	42	113	LM (+)	4	4
	LM (−)	3123	79	LM (−)	317	1
RF	LM (+)	46	109	LM (+)	3	5
	LM (−)	3136	66	LM (−)	318	0
GNB	LM (+)	32	123	LM (+)	0	8
	LM (−)	3051	151	LM (−)	313	5
KNN	LM (+)	49	106	LM (+)	4	4
	LM (−)	3111	91	LM (−)	316	2
MLP	LM (+)	64	91	LM (+)	5	3
	LM (−)	3131	71	LM (−)	303	15
CART	LM (+)	41	114	LM (+)	3	5
	LM (−)	3100	102	LM (−)	313	5
SVM	LM (+)	35	120	LM (+)	0	8
	LM (−)	3059	143	LM (−)	293	25
Stacking	LM (+)	26	129	LM (+)	0	8
	LM (−)	3062	140	LM (−)	303	15

*LM* liver metastasis, *LGBM* Light Gradient Boosting Decision, *RF* Random Forest, *GNB* Gaussian Naive Bayesian, *KNN* K-Nearest Neighbor, *MLP* Multilayer Perceptron, *CART* Classification and Regression Trees, *SVM* Support Vector Machine

To further assess comprehensive performance of the AI model, we made comparisons between previous models and logistic regression ones based on our data. Corresponding results testified that the stack-bagging model outperformed other models (Additional file 6: Table S5).

Furthermore, by means of employing survival status and time from the SEER database, we plotted the Kaplan Meier (K–M) curves of the testing set. It was universally acknowledged that LM functioned as an unfavorable prognostic indicator for CRC patients (Additional file 7: Figure S2a). Likewise, we found that the stacking model resembled LM in predicting T1 CRC patients’ outcomes (Additional file 7: Figure S2b).

### Comparison of significance of each factor

In all single models, tumor size, preoperative CEA levels, tumor deposits, N stage, histology, and PNI all revealed equally fundamental significance in predicting for LM in T1 CRC. Despite the fact that the AI model manifested desirable performance, the individualized influence of each factor on the result and underlying relationships between these factors remained largely unknown. Hence, we calculated and digitized the significance of each factor used in the built-up AI models (Additional file 8: Figure S3 and Additional file 9: Table S6). Coinciding with previous anticipation, we found that tumor size, CEA level prior to surgery, tumor deposits, and N stage were the top four crucial predictors among all models. Particularly worth mentioning is the fact that tumor size standed out as the most critical one amidst nearly all models.

### Subgroup analysis

**Fig. 3:**
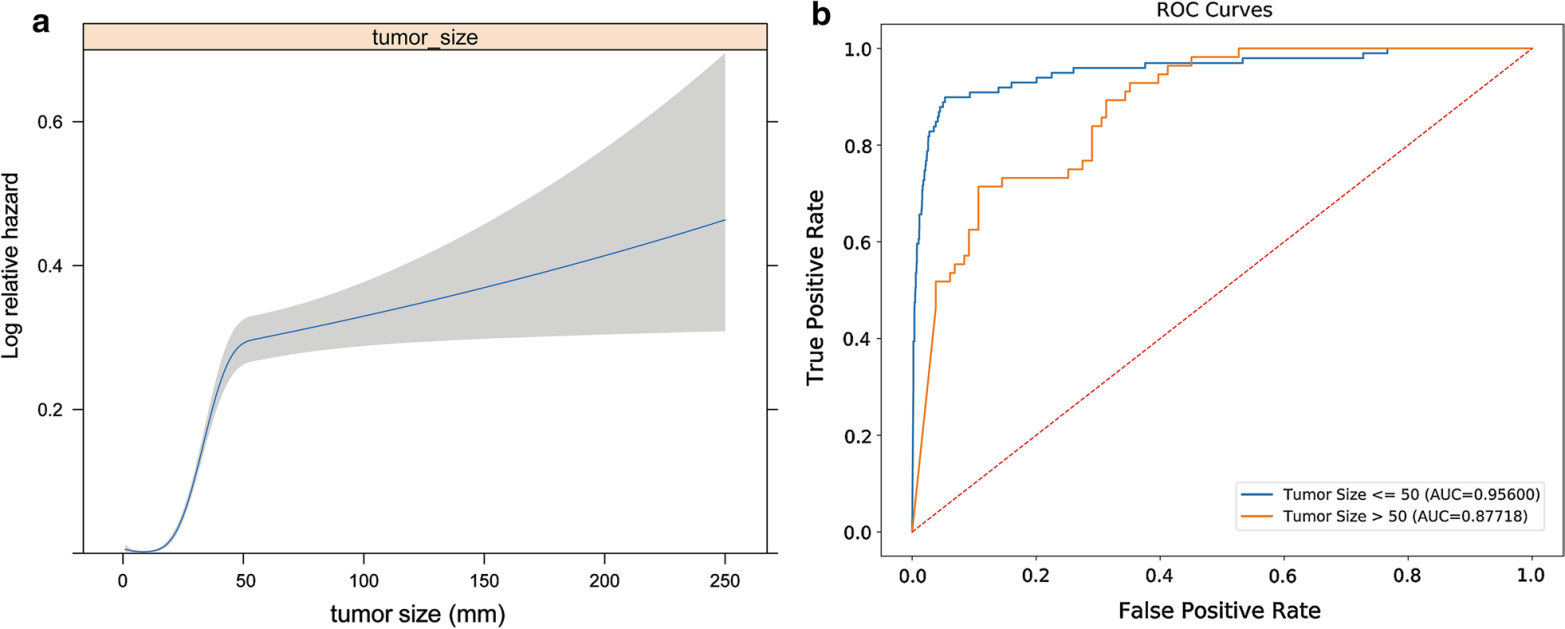
Estimation of models’ discriminant capability for T1 CRC patients with different tumor sizes. **a** Restricted cubic spline of tumor size. **b** ROC curves of seven individual models and stacking model for patients with different tumor sizes (1–50 mm and > 50 mm). CRC: colorectal cancer; and ROC: receiver operating characteristic

On account of the reality that tumor size might play a dominant role in prediction while other parameters made relatively less contributions in terms of forecasting model performance, we determined to further investigate the association of tumor size with LM hazard. Firstly, RCS function of tumor size in the training set exhibited a non-linear profile (non-linearity P value < 0.001; Fig. 3a), indicating that this clinical feature should be encoded as a categorical factor and was inappropriate for being employed in canonical logistic regression analysis. Notably, the 50 mm tumor size demonstrated an optimal cut-off value for subgroup analysis (Fig. 3a). Therefore, we utilized the representative AUC and AP value to further explore the model performance in disparate subgroups. Analysis results indicated that AUC values of 1–50 mm and > 50 mm subgroups reached 0.956 and 0.8772 respectively (Fig. 3b).

In light of the fact that patients with tumor size larger than 50 mm accounted for a lower percentage than the 1–50 mm subgroup, we further divided patients into 1–10 mm and 1–20 mm subgroups. The AUC values (Bagging Stacking model) of 1–10 mm and 1–20 mm subgroups reached 0.8212 and 0.8608 respectively (Additional file 10: Figure S4a and b). Generally speaking, the stacking model was triumphantly verified to possess a favorable prediction capacity in T1 CRC patients with small tumor sizes.

### Clinical application

**Fig. 4:**
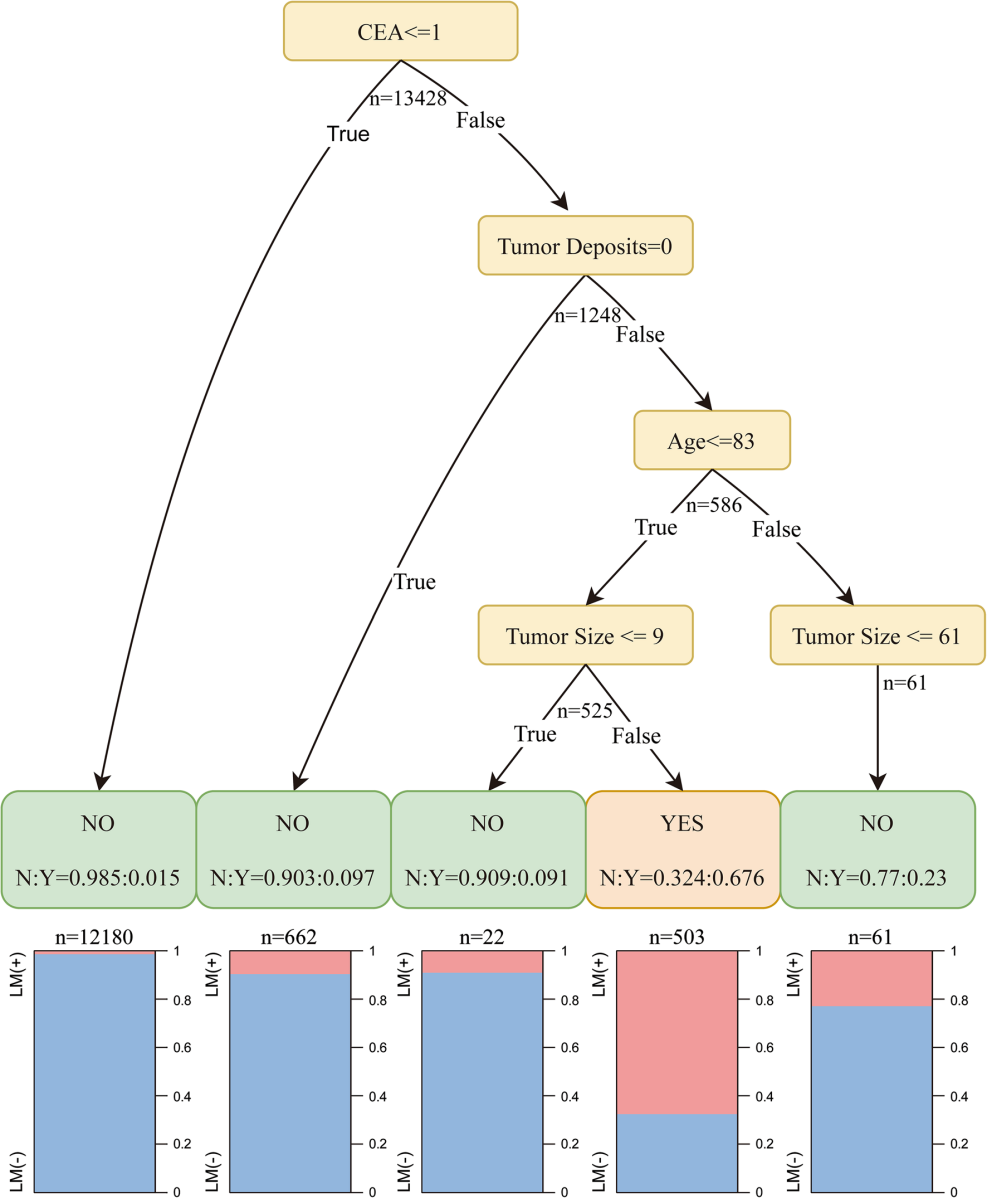
Decision tree tool to discriminate liver metastasis in T1 colorectal cancer patients

Although the stacking model manifested both desirable and robust predictive power for LM in T1 CRC, the model was intricate in nature which could not be easily apprehended by clinicians. As a consequence, we developed an easy-to-use instrument (clinical decision tree) for the sake of supplementing clinical decision-making process with pragmatic guidance (Fig. 4). In this decision tree, target population were categorized into five groups according to the following four most crucial factors namely CEA level, tumor size, tumor deposits and age. The ROC of clinical decision tree archived 0.949 (Additional file 11: Figure S5), undoubtedly a demonstration of its remarkable discriminative and predictive ability. The population harboring such characteristics as CEA Positive or Borderline, positive tumor deposits, age ≤ 83 and tumor size > 10 manifested high proportion of LM (32.4%) and could be categorized into the high-risk subgroup of LM. On the contrary, remanent three types of patients uniformly demonstrated low occurrence of LM.

## Discussion

Liver is generally identified as one of the most commonly seen metastasis sites for CRC while LM is universally recognized as the most lethal factor of CRC patients [36, 37]. Early diagnosis of LM could assist clinicians in taking prompt and timely intervention to improve the prognosis of patients, especially for CRC T1 patients [38, 39]. CRC patients in T1 stage could select either surgical or endoscopic treatment, partly depending on the status of distant metastasis. Hence, a convenient and accurate predictive model of LM is urgently demanded to offer guidance on personalized therapeutic strategies.

In the study, we established an innovative and convenient model to predict early LM by incorporating **11** clinicopathologic parameters in T1 CRC utilizing seven AI methods. We firstly combined our real-world researches with public data online on a large scale to comprehensively construct and assess LM predictive models in T1 CRC. Given that the AUC of these models was more extensive than 0.94 and model accuracy was approximately as 100% as possible, we came to the conclusion that above-established models were desirable and robust in yielding favorable clinical benefits, which might be of tremendous assistance to clinicians in the selection process of underlying LM CRC patients. More intriguingly, our model manifested extraordinary competence indiscriminating the LM in T1 CRC patients with small tumor size (1–50 mm) from others. Ultimately, to develop an easy-to-use instrument in clinical practice, we plotted a decision tree to screen out the high-risk population of LM. The visualized decision tree was not only precise but also easy to comprehend for clinicians.

Our real-world research incorporated 326 cases of T1 CRC, amidst which LM occurred in merely eight patients (8/326), significantly lower than that of the SEER database (762/16785, P < 0.001). The discrepancy in the LM ratio might be attributed to low diagnostic efficacy in developing countries [40, 41]. Interestingly, compared with more advanced T stage CRC patients (169/326), PNI was more frequently appeared in T1 CRC patients of our hospital (1266/8226), consistent with results of the SEER database (11350/16785). Abundant evidence has demonstrated that the percentage of PNI occurring in all T stages is approximately 10–15%. Moreover, PNI is an independent biomarker that indicates aggressive behavior and unfavorable prognosis of CRC [42–45]. Nonetheless, scarcely explained by published literature were underlying causes behind the high ratio of PNI in T1 CRC which deserved further investigation. In addition, serum CEA was confirmed to have a positive relationship with LM. Accumulating evidence has suggested that the expression level of CEA could function as an independent indicator for the prognosis of CRC patients [46]. Therefore, it was not surprising that the concentration of preoperative plasma CEA was significantly higher in CRC patients with LM compared with those with primary CRC [47–49]. Besides, among all indicators, tumor size has been regarded as one of the most significant biomarkers in predicting LM status. It has been reported that tumor size was intimately associated with both lymph and hepatic metastases of CRC [50]. Furthermore, scientists have verified that age might play a nonnegligible role in the advancement and prognosis of CRC [51]. Despite increment in young CRC patients, compelling evidence revealed that the young tended to have more favorable outcomes than the old [51]. Contradictorily, our research indicated that CRC patients younger than 60 years of age were more apt to experience risk of LM than their counterparts, which was consistent with several other researchers [52–54]. The probable reason might have something to do with frequently occurred mismatch repair gene mutation and upregulated aggressive neoplastic biology in younger patients [55].

To date, multitudes of investigators have constructed diverse models to predict the metastatic capability of CRC. For instance, Tang et al. [14] built up a novel nomogram to forecast LM in all T stages CRC patients via utilizing multivariable Cox regression. They also found that synchronous LM was an independent prognostic factor for CRC patients. Analogously, Li et al. [56] employed the SEER database to construct a T1 CRC all distant metastasis model by virtue of the conventional logistic regression. Howbeit, due to the limitation of the algorithm and the approach to process data, they acquired a passable model (AUC = 0.879) with ineluctable overfitting. Recently, with enormous technical advancement of AI, the application of ML model in neoplastic diagnosis and prognostic assessment has become increasingly prevalent [57, 58]. Numerous novel ML algorithms have remedied deficiencies of canonical statistical methods, such as overfitting, unbalanced data distribution and so on. Ji Hyun Ahn et al. [19] developed an innovative model (AUC = 0.96) to predict LNM in the early stage of CRC patients via utilizing the SEER database and adopting seven AI methods. Nevertheless, these studies were retrospective, single-center, and with small quantities of patients. Additionally, Ichimasa et al. [59] testified that AI could downregulate unnecessary surgery after endoscopic resection of LNM (−) T1 CRC compared with current guidelines. Nonetheless, few models for predicting the incidence of LM in T1 CRC patients were developed and assessed utilizing AI methods. In the current study, we established nine models and then validated them in our own dataset. Besides, their efficacy of predicting LM in early CRC was also compared by dint of easily available clinical and histopathological features. Moreover, we found that our constructed AI models could not only assist clinicians in selecting patients with a high risk of LM, but also resemble LM in accurately predicting T1 CRC patients’ outcomes. Our models still exhibited a superior ability to discriminate the LM in T1 CRC patients with small tumor size from others (1–50 mm).

So far, only surgical resection has been verified as a curative therapeutic approach for CRC patients with early and resectable LM [60, 61]. For patients with untestable LM, early application of systemic chemotherapy might ameliorate the prognosis and enhance the median survival ratio [62]. Integrating entire above-mentioned results, we believed that further utilization of T1 CRC LM models would contribute to the clinical decision making and improve the present therapeutic status.

Admittedly, there still exists several limitations and weaknesses in the study. Firstly, in light that the SEER database is an open and available national program of America, these newly established models might not work in other countries. Secondly, quantities of enrolled patients in our hospital were far from sufficient, and merely eight patients manifested LM status. These shortcomings might lead to a limited verification outcome. In the future, more in-depth and extensive studies will be urgently needed. In addition, we intend to package the stacking model and decision tree to a novel software or website and validate them clinically afterwards in our next work.

## Conclusions

In the present study, we successfully established an innovative and stacking bagging model which incorporates **11** clinicopathologic features to predict the incidence of LM in T1 CRC. Our findings indicated that age, gender, married status, primary site, tumor size, CEA, tumor type, grade, N stage and PNI were crucial factors for forecasting LM, amidst which tumor size mattered most. As expected, the stacking bagging model, which integrated strengths of seven single models, demonstrated the strongest predictive power in both databases of SEER and our hospital. Moreover, we found that the stacking model resembled LM when it came to accurate prediction of T1 CRC patients’ outcomes. A novel easy-to-use tool (decision tree) was developed to guide clinicians in screening out high-risk patients of LM and exposing them to more aggressive therapeutic strategies.

## Supplementary Information

**Additional file 1: Table S1.** Normalization standards of clinical data in outer validation set.

**Additional file 2: Table S2.** References for property values of clinical features in models.

**Additional file 3: Figure S1. **PR curves for overall models. Inner validation in SEER database: **(a)** PR curves, indicating the tradeoff between precision and recall. Outer validation in our Chinese cohort: **(b)** PR curves, indicating the tradeoff between precision and recall. SEER: Surveillance, Epidemiology, and End Results; and PR: precision-recall.

**Additional file 4: Table S3.** Performance of developed models in inner datasets.

**Additional file 5: Table S4.** Performance of developed models in our real-world dataset.

**Additional file 6: Table S5.** Comparison of AI algorithms and logistic regression algorithm.

**Additional file 7: Figure S2. **Evaluation of the prognostic value for stacking-bagging model. **(a)** The survival curve based real data. **(b)** The survival curve based on predictive outcomes.

**Additional file 8: Figure S3.** Factor importance of the developed models. Bar graphs describe the proportion of importance of the different predictors in models. The top ten factor importance were exhibited in models: **(a)** Average of factor importance in seven models, **(b)** LGBM, **(c)** RF, **(d)** GNB, **(e)** KNN, **(f)** MLP, **(g)** CART, and **(h)** SVM. LGBM: Light Gradient Boosting Decision; RF: Random Forest; GNB: Gaussian Naive Bayesian; KNN: k-nearest neighbor algorithm; MLP: Multilayer Perceptron; CART: Classification and Regression Trees; and SVM: Support Vector Machine.

**Additional file 9: Table S6. **Significances of clinical features in AI models.

**Additional file 10: Figure S4.** Models’ prediction value for T1 CRC patients with small tumor sizes. **(a)** ROC curves of seven individual models and stacking model in tumor size (1–10 mm). **(b)** ROC curves of seven individual models and stacking model in tumor size (1–20 mm).

**Additional file 11: Figure S5.** Performance of decision tree model. **(a)** ROC curves of seven individual models and stacking model. **(b)** PR curves, indicating the tradeoff between precision and recall. ROC: receiver operating characteristic; and PR: precision-recall.

**Additional file 12:** Original data of inner testing set of SEER.

**Additional file 13:** Original data of inner training set of SEER.

**Additional file 14:** Original data of outer validation set from Xijing hospital.

## Data Availability

The datasets used and/or analyzed during the current study are included in this published article and its additional files.

## Abbreviations

CRC: Colorectal cancer
LM: Liver metastasis
OS: Overall survival
AI: Artificial intelligence
ML: Machine learning
SEER: Surveillance, Epidemiology, and End Results
LNM: Lymph node metastasis
CEA: Carcinoembryonic antigen
PNI: Perineural invasion
LGBM: Light Gradient Boosting Decision
RF: Random Forest
CART: Classification and Regression Trees
KNN: K-Nearest Neighbor
SVM: Support Vector Machine
GNB: Gaussian Naive Bayesian
MLP: Multilayer Perceptron
Bagging: Bootstrap aggregating
AUC: Area under the curve
NPV: Negative predictive value
FDR: False discovery rate
AP: Average precision
AU-ROC: Area under receiver operating characteristic curves
PR: Precision-recall
RCS: Restricted cubic splines
FN: False Negative
FP: False Positive
K-M: Kaplan Miere
