# Supplementary material for: Application of artificial intelligence in a real-world research for predicting the risk of liver metastasis in T1 colorectal cancer
Source: Cancer Cell Int. 2022 Jan 15;22:28. doi: 10.1186/s12935-021-02424-7 (PMC8761313; doi:10.1186/s12935-021-02424-7)
Supplement: Supplementary file 1 — Additional file 1: Table S1. Normalization standards of clinical data in outer validation set. [file 12935_2021_2424_MOESM1_ESM.docx]

Table S1. Normalization standards of clinical data in outer validation set.

| **Clinical data** | **Normalization Standard** |
| --- | --- |
| **Basic demographic data** |  |
| Age | Age at diagnosis. |
| Marital status | Divided into seven categories: Married (including common law), Single (never married), Widowed, Divorced, Unknown, Separated,  and Unmarried or Domestic Partner. |
| **Tumor information** | (Checked by experienced pathologists and enterologists) |
| Tumor size | the largest diameter of the primary tumor. |
| Histology and  Primary site | According to collaborative stage data collection system coding  instructions (Appendix 6) of SEER database. |
| TNM stage | According to AJCC 7th edition. |
| Tumor grade | According to AJCC 7th edition divided into four categories:  Well differentiated, Moderately differentiated, Poorly differentiated, and Undifferentiated. |
| **Laboratory index** | (Checked by experienced medical technologists and enterologists) |
| CEA | Examined prior to surgery. Divided into three categories:  Positive (Nonsmoker: >2.5ng/ml, Smoker: >5ng/ml), Borderline (Nonsmoker: 2.5ng/ml, Smoker: 5ng/ml), Negative (Nonsmoker: <2.5ng/ml, Smoker: <5ng/ml). |
| tumor deposits | According to AJCC 7th edition. Divided into two categories:  Tumor deposits identified, and No tumor deposits. |
| Perineural Invasion | According to AJCC 7th edition. Divided into two categories:  Yes and No. |

SEER, Surveillance, Epidemiology, and End Results; AJCC, American Joint Committee on Cancer; CEA, carcinoembryonic antigen.
