# Supplementary material for: Application of artificial intelligence in a real-world research for predicting the risk of liver metastasis in T1 colorectal cancer
Source: Cancer Cell Int. 2022 Jan 15;22:28. doi: 10.1186/s12935-021-02424-7 (PMC8761313; doi:10.1186/s12935-021-02424-7)
Supplement: Supplementary file 2 — Additional file 2: Table S2. References for property values of clinical features in models. [file 12935_2021_2424_MOESM2_ESM.docx]

Table S2 References for property values of clinical features in models.

| **Variables** |  | **Property Values** |  |
| --- | --- | --- | --- |
|  |  |  |  |
| **Gender** |  |  |  |
| Male |  | 0 |  |
| Female |  | 1 |  |
| **Race** |  |  |  |
| White |  | 0 |  |
| Black |  | 1 |  |
| Asian or Pacific Islander |  | 2 |  |
| American Indian/Alaska Native |  | 3 |  |
| **Marital Status** |  |  |  |
| Married (including common law) |  | 0 |  |
| Single (never married) |  | 1 |  |
| Widowed |  | 2 |  |
| Divorced |  | 3 |  |
| Unknown |  | 4 |  |
| Separated |  | 5 |  |
| Unmarried or Domestic Partner |  | 6 |  |
| **Primary Site** |  |  |  |
| C20.9-Rectum, NOS |  | 0 |  |
| C18.7-Sigmoid colon |  | 1 |  |
| C18.2-Ascending colon |  | 2 |  |
| C18.0-Cecum |  | 3 |  |
| C18.1-Appendix |  | 4 |  |
| C19.9-Rectosigmoid junction |  | 5 |  |
| C18.4-Transverse colon |  | 6 |  |
| C18.6-Descending colon |  | 7 |  |
| C18.3-Hepatic flexure of colon |  | 8 |  |
| C18.5-Splenic flexure of colon |  | 9 |  |
| C18.9-Colon, NOS |  | 10 |  |
| C18.8-Overlapping lesion of colon |  | 11 |  |
| **Grade** |  |  |  |
| Moderately differentiated; Grade II |  | 0 |  |
| Well differentiated; Grade I |  | 1 |  |
| Poorly differentiated; Grade III |  | 2 |  |
| Undifferentiated; anaplastic; Grade IV |  | 3 |  |
| **Histology** |  |  |  |
| 8140/3: Adenocarcinoma, NOS |  | 0 |  |
| 8263/3: Adenocarcinoma in tubulovillous adenoma |  | 1 |  |
| 8210/3: Adenocarcinoma in adenomatous polyp |  | 2 |  |
| 8240/3: Carcinoid tumor, NOS |  | 3 |  |
| 8261/3: Adenocarcinoma in villous adenoma |  | 4 |  |
| 8246/3: Neuroendocrine carcinoma, NOS |  | 5 |  |
| 8480/3: Mucinous adenocarcinoma |  | 6 |  |
| 8070/3: Squamous cell carcinoma, NOS |  | 7 |  |
| 8249/3: Atypical carcinoid tumor |  | 8 |  |
| 8490/3: Signet ring cell carcinoma |  | 9 |  |
| 8481/3: Mucin-producing adenocarcinoma |  | 10 |  |
| 8211/3: Tubular adenocarcinoma |  | 11 |  |
| 8010/3: Carcinoma, NOS |  | 12 |  |
| 8936/3: Gastrointestinal stromal sarcoma |  | 13 |  |
| 8041/3: Small cell carcinoma, NOS |  | 14 |  |
| 8262/3: Villous adenocarcinoma |  | 15 |  |
| 8245/3: Adenocarcinoid tumor |  | 16 |  |
| 8243/3: Goblet cell carcinoid |  | 17 |  |
| 8213/3: Serrated adenocarcinoma |  | 18 |  |
| 8510/3: Medullary carcinoma, NOS |  | 19 |  |
| 8083/3: Basaloid squamous cell carcinoma |  | 20 |  |
| 8255/3: Adenocarcinoma with mixed subtypes |  | 21 |  |
| 8071/3: Squamous cell carcinoma, keratinizing, NOS |  | 22 |  |
| 8013/3: Large cell neuroendocrine carcinoma |  | 23 |  |
| 8560/3: Adenosquamous carcinoma |  | 24 |  |
| 8244/3: Mixed adenoneuroendocrine carcinoma (ICD-O-3 update) |  | 25 |  |
| 8241/3: Enterochromaffin cell carcinoid |  | 26 |  |
| 8072/3: Squamous cell carcinoma, large cell, nonkeratinizing, NOS |  | 27 |  |
| 8000/3: Neoplasm, malignant |  | 28 |  |
| 8470/3: Mucinous cystadenocarcinoma, NOS |  | 29 |  |
| 8574/3: Adenocarcinoma with neuroendocrine differentiation |  | 30 |  |
| 8260/3: Papillary adenocarcinoma, NOS |  | 31 |  |
| 8144/3: Adenocarcinoma, intestinal type |  | 32 |  |
| 8051/3: Verrucous carcinoma, NOS |  | 33 |  |
| 8310/3: Clear cell adenocarcinoma, NOS |  | 34 |  |
| 8032/3: Spindle cell carcinoma, NOS |  | 35 |  |
| 8507/3: Ductal carcinoma, micropapillary |  | 36 |  |
| 8020/3: Carcinoma, undifferentiated, NOS |  | 37 |  |
| 8380/3: Endometrioid carcinoma |  | 38 |  |
| 8004/3: Malignant tumor, spindle cell type |  | 39 |  |
| 8323/3: Mixed cell adenocarcinoma |  | 40 |  |
| 8082/3: Lymphoepithelial carcinoma |  | 41 |  |
| 8073/3: Squamous cell carcinoma, small cell, nonkeratinizing |  | 42 |  |
| 8550/3: Acinar cell carcinoma |  | 43 |  |
| 8012/3: Large cell carcinoma, NOS |  | 44 |  |
| 8052/3: Papillary squamous cell carcinoma |  | 45 |  |
| **N** |  |  |  |
| N0 |  | 0 |  |
| N1 |  | 1 |  |
| N2 |  | 2 |  |
| **CEA** |  |  |  |
| NA |  | 0 |  |
| Negative |  | 1 |  |
| Positive |  | 2 |  |
| Borderline |  | 3 |  |
| **Tumor Deposits** |  |  |  |
| No tumor deposits |  | 0 |  |
| NA |  | 1 |  |
| Tumor Deposits identified |  | 2 |  |
| **Perineural Invasion** |  |  |  |
| Yes |  | 0 |  |
| NA |  | 1 |  |
| No |  | 2 |  |

CEA, carcinoembryonic antigen; NOS: not otherwise specified.
