# Supplementary material for: Application of artificial intelligence in a real-world research for predicting the risk of liver metastasis in T1 colorectal cancer
Source: Cancer Cell Int. 2022 Jan 15;22:28. doi: 10.1186/s12935-021-02424-7 (PMC8761313; doi:10.1186/s12935-021-02424-7)
Supplement: Supplementary file 4 — Additional file 4: Table S3. Performance of developed models in inner datasets. [file 12935_2021_2424_MOESM4_ESM.docx]

Table S3: Performance of developed models in inner datasets.

| **Models** | **AUC** | **Sensitivity** | **Specificity** | **Precision** | **NPV** | **FDR** | **Accuracy** | **AP** | **F1** | **MCC** |
| --- | --- | --- | --- | --- | --- | --- | --- | --- | --- | --- |
| LGBM | 0.9608 | 0.7677 | 0.9753 | 0.6010 | 0.9886 | 0.3990 | 0.9657 | 0.7150 | 0.6742 | 0.6619 |
| RF | 0.9589 | 0.7226 | 0.9744 | 0.5773 | 0.9864 | 0.4227 | 0.9628 | 0.7051 | 0.6418 | 0.6268 |
| GNB | 0.9504 | 0.7935 | 0.9535 | 0.4522 | 0.9896 | 0.5478 | 0.9461 | 0.4981 | 0.5761 | 0.5745 |
| KNN | 0.9520 | 0.6839 | 0.9719 | 0.5408 | 0.9845 | 0.4592 | 0.9586 | 0.6244 | 0.6040 | 0.5869 |
| MLP | 0.9443 | 0.6000 | 0.9778 | 0.5671 | 0.9806 | 0.4329 | 0.9604 | 0.5788 | 0.5831 | 0.5625 |
| CART | 0.9558 | 0.7806 | 0.9685 | 0.5450 | 0.9892 | 0.4550 | 0.9598 | 0.6819 | 0.6419 | 0.6326 |
| SVM | 0.9422 | 0.7677 | 0.9544 | 0.4491 | 0.9884 | 0.5509 | 0.9458 | 0.5524 | 0.5667 | 0.5620 |
| Stacking | 0.9631 | 0.8452 | 0.9566 | 0.4852 | 0.9922 | 0.5148 | 0.9514 | 0.6927 | 0.6165 | 0.6187 |

AUC, area under curve; NPV, negative predictive value; FDR, false discovery rate; AP, average precision; MCC, matthews correlation coefficient; LGBM, Light Gradient Boosting Decision; RF, Random Forest; GNB, Gaussian Naive Bayesian; KNN, K-Nearest Neighbor; MLP, Multilayer Perceptron; CART, Classification and Regression Trees; SVM, Support Vector Machine.
