# Supplementary material for: Application of artificial intelligence in a real-world research for predicting the risk of liver metastasis in T1 colorectal cancer
Source: Cancer Cell Int. 2022 Jan 15;22:28. doi: 10.1186/s12935-021-02424-7 (PMC8761313; doi:10.1186/s12935-021-02424-7)
Supplement: Supplementary file 5 — Additional file 5: Table S4. Performance of developed models in our real-world dataset. [file 12935_2021_2424_MOESM5_ESM.docx]

Table S4 Performance of developed models in our real-world dataset.

| Models | AUC | Sensitivity | Specificity | Precision | NPV | FDR | Accuracy | AP | F1-Score | MCC |
| --- | --- | --- | --- | --- | --- | --- | --- | --- | --- | --- |
| LGBM | 0.9882 | 0.7500 | 1.0000 | 1.0000 | 0.9938 | 0.0000 | 0.9939 | 0.8864 | 0.8571 | 0.8633 |
| RF | 0.9917 | 0.6250 | 1.0000 | 1.0000 | 0.9907 | 0.0000 | 0.9908 | 0.8596 | 0.7692 | 0.7869 |
| GNB | 0.9906 | 0.8750 | 0.9843 | 0.5833 | 0.9968 | 0.4167 | 0.9816 | 0.6407 | 0.7000 | 0.7061 |
| KNN | 0.9336 | 0.6250 | 0.9937 | 0.7143 | 0.9906 | 0.2857 | 0.9847 | 0.6281 | 0.6667 | 0.6604 |
| MLP | 0.8247 | 0.2500 | 0.9560 | 0.1250 | 0.9806 | 0.8750 | 0.9387 | 0.0873 | 0.1667 | 0.1475 |
| CART | 0.9969 | 0.8750 | 0.9937 | 0.7778 | 0.9968 | 0.2222 | 0.9908 | 0.9152 | 0.8235 | 0.8203 |
| SVM | 0.9894 | 0.8750 | 0.9465 | 0.2917 | 0.9967 | 0.7083 | 0.9448 | 0.8147 | 0.4375 | 0.4867 |
| Stacking | 0.9917 | 0.8750 | 0.9623 | 0.3684 | 0.9967 | 0.6316 | 0.9601 | 0.8113 | 0.5185 | 0.5529 |

AUC, area under curve; NPV, negative predictive value; FDR, false discovery rate; AP, average precision; MCC, matthews correlation coefficient; LM, liver metastasis; LGBM, Light Gradient Boosting Decision; RF, Random Forest; GNB, Gaussian Naive Bayesian; KNN, K-Nearest Neighbor; MLP, Multilayer Perceptron; CART, Classification and Regression Trees; SVM, Support Vector Machine.
