# Supplementary material for: Application of artificial intelligence in a real-world research for predicting the risk of liver metastasis in T1 colorectal cancer
Source: Cancer Cell Int. 2022 Jan 15;22:28. doi: 10.1186/s12935-021-02424-7 (PMC8761313; doi:10.1186/s12935-021-02424-7)
Supplement: Supplementary file 6 — Additional file 6: Table S5. Comparison of AI algorithms and logistic regression algorithm. [file 12935_2021_2424_MOESM6_ESM.docx]

Table S5 Comparison of AI algorithms and logistic regression algorithm.

| **Model** | **Formula** | **AUC** |
| --- | --- | --- |
| Stack-bagging (AI algorithm) | - | **0.963** |
| Logistic regression with  data normalization by AI algorithm | e^(-5.55615136-0.09747314*Gender+1.26878795*Race+2.77946385*Primary Site -0.49524628*Grade-0.17059261*Histology-0.52411816*N+0.64749917*Tumor Deposits +2.30944488*CEA+0.66099821*Perineural Invasion+0.16846474*Tumor Size +1.50292232*Age+1.34062037*Marital status) | **0.903** |
| Logistic regression without  data normalization by AI algorithm | e^(-5.29927833-0.0231653*Gender-0.03917969*Race-0.00994608*Primary Site -0.05932806*Grade-0.06884165*Histology+0.9485313*N+2.12589245*Tumor Deposits +1.48046876*CEA+0.60305853*Perineural Invasion+0.03530818*Tumor Size -0.00314196*Age-0.05781427*Marital status) | **0.878** |
| Logistic regression in previous  research about T1 all distant metastasis | e^(-5.81-0.30*age(≥60)+1.36*tumor size(>5 cm)+0.53*tumor size(unknown) +2.70*CEA(positive)-0.2*CEA(unknown)+1.42*mucinous adenocarcinoma +1.78*Signet ring cell carcinoma+0.98*tumor deposit(positive)+1.13*tumor deposit (unknown)+0.62*poorly or un-differentiated+0.43*unknown differentiation +0.92*perineural invasion(positive)+0.74*perineural invasion(unknown)+1.39*N stage(N1) +2.14*N stage(N2)+1.11*unknown N stage) | **0.899** |

AI, artificial intelligence; CEA, carcinoembryonic antigen.
