# Supplementary figures and images for: Application of artificial intelligence in a real-world research for predicting the risk of liver metastasis in T1 colorectal cancer
Source: Cancer Cell Int. 2022 Jan 15;22:28. doi: 10.1186/s12935-021-02424-7 (PMC8761313; doi:10.1186/s12935-021-02424-7)

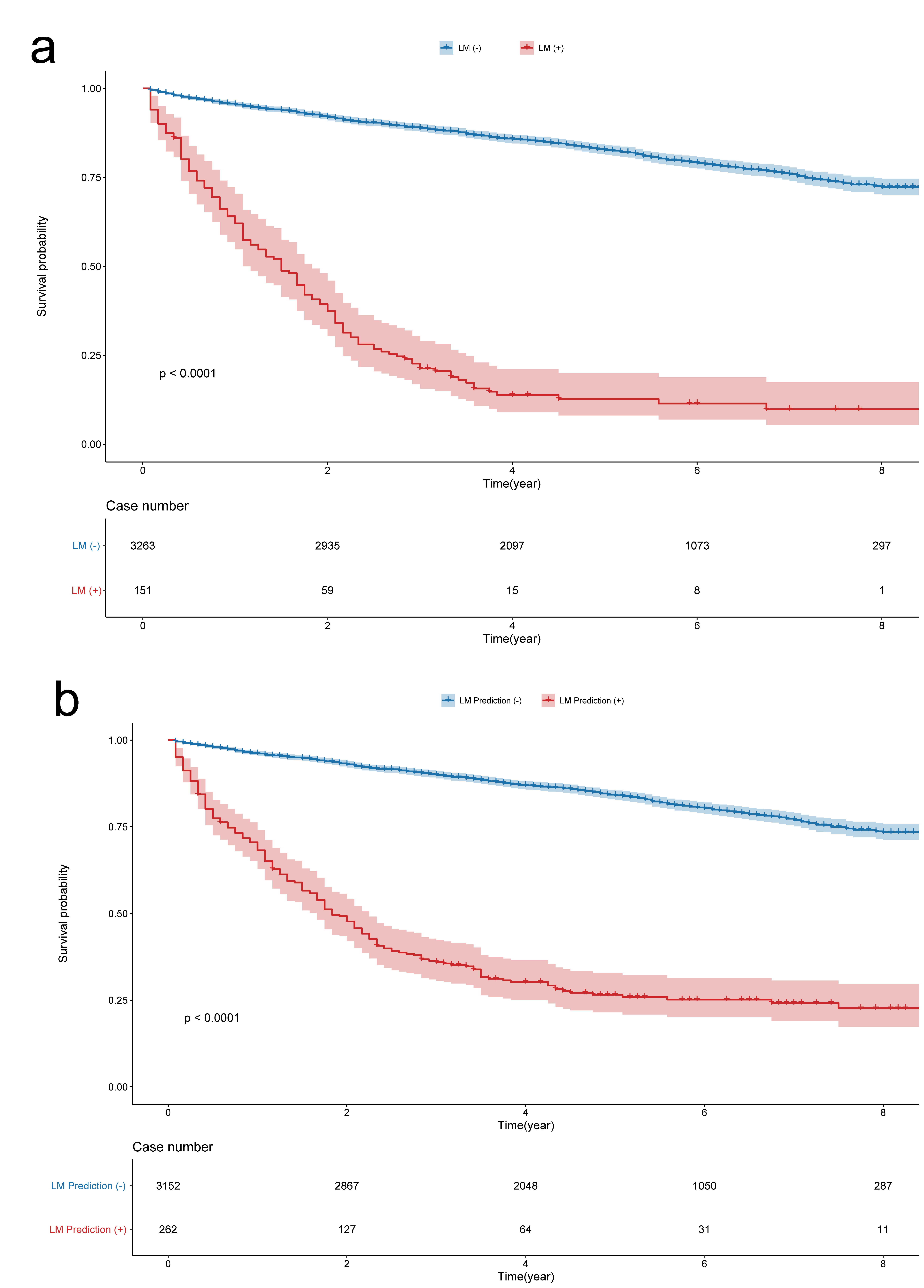

Supplement: Supplementary file 7 — Additional file 7: Figure S2. Evaluation of the prognostic value for stacking-bagging model. (a) The survival curve based real data. (b) The survival curve based on predictive outcomes. [file 12935_2021_2424_MOESM7_ESM.tif]

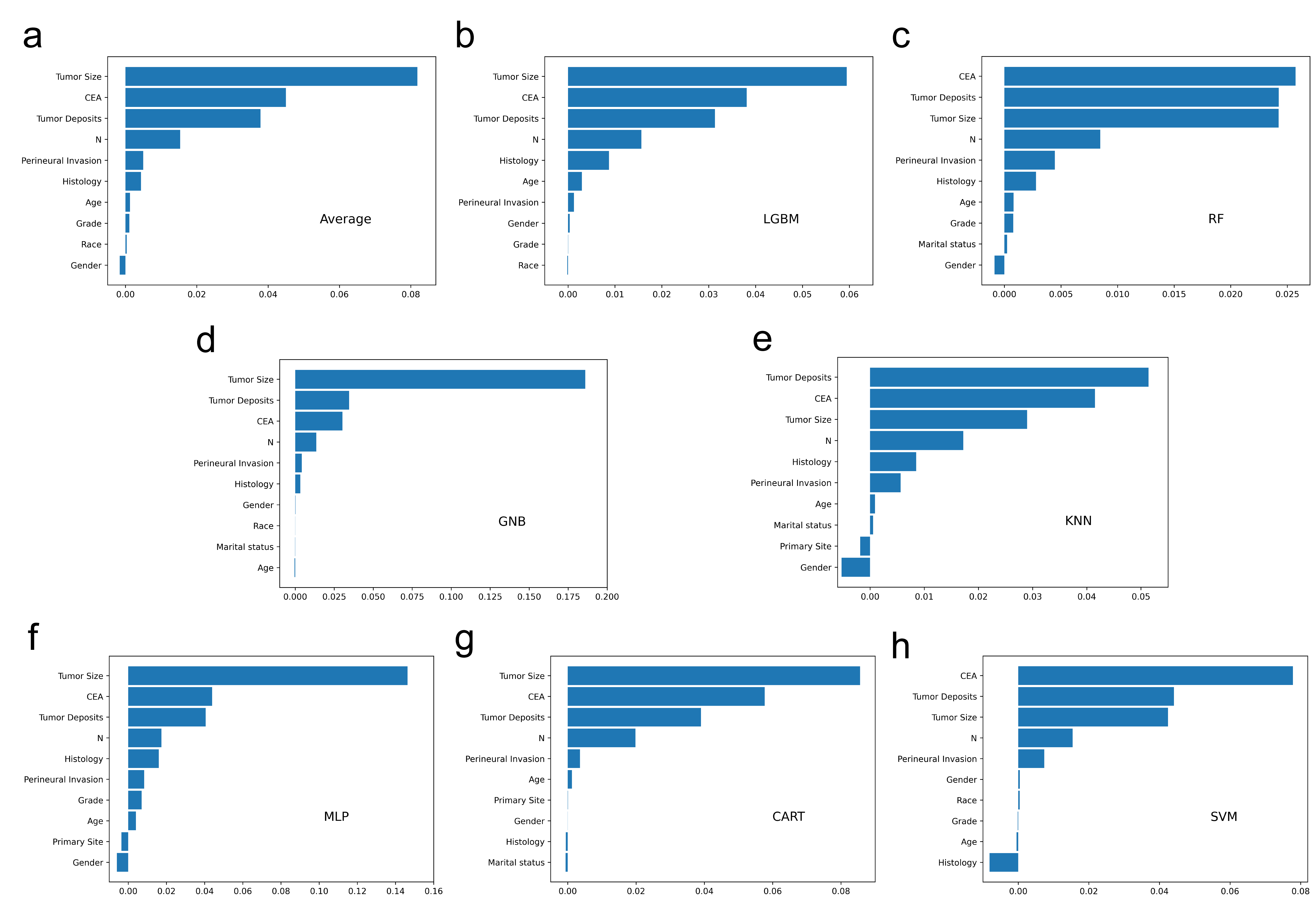

Supplement: Supplementary file 8 — Additional file 8: Figure S3. Factor importance of the developed models. Bar graphs describe the proportion of importance of the different predictors in models. The top ten factor importance were exhibited in models: (a) Average of factor importance in seven models, (b) LGBM, (c) RF, (d) GNB, (e) KNN, (f) MLP, (g) CART, and (h) SVM. LGBM: Light Gradient Boosting Decision; RF: Random Forest; GNB: Gaussian Naive Bayesian; KNN: k-nearest neighbor algorithm; MLP: Multilayer Perceptron; CART: Classification and Regression Trees; and SVM: Support Vector Machine. [file 12935_2021_2424_MOESM8_ESM.tif]
