# Supplementary material for: Application of artificial intelligence in a real-world research for predicting the risk of liver metastasis in T1 colorectal cancer
Source: Cancer Cell Int. 2022 Jan 15;22:28. doi: 10.1186/s12935-021-02424-7 (PMC8761313; doi:10.1186/s12935-021-02424-7)
Supplement: Supplementary file 9 — Additional file 9: Table S6. Significances of clinical features in AI models. [file 12935_2021_2424_MOESM9_ESM.docx]

Table S6 Significances of clinical features in AI models.

| **Model** | **Tumor size** | **CEA** | **Tumor Deposits** | **N** | **Perineural Invasion** | **Histology** | **Age** | **Grade** | **Marital Status** | **Race** | **Primary Site** | **Gender** |
| --- | --- | --- | --- | --- | --- | --- | --- | --- | --- | --- | --- | --- |
| LGBM | 0.0582 | 0.0333 | 0.0249 | 0.0102 | 0.0030 | 0.0037 | 0.0014 | 0.0003 | -0.0002 | -0.0007 | 0.0005 | 0.0001 |
| RF | 0.0229 | 0.0257 | 0.0198 | 0.0081 | 0.0038 | 0.0030 | 0.0012 | 0.0012 | 0.0002 | 0.0000 | -0.0001 | -0.0004 |
| GNB | 0.0251 | 0.0598 | 0.0445 | 0.0224 | 0.0039 | 0.0050 | 0.0006 | 0.0002 | 0.0002 | 0.0001 | 0.0000 | 0.0008 |
| KNN | 0.0263 | 0.0399 | 0.0359 | 0.0163 | 0.0051 | 0.0103 | 0.0024 | 0.0005 | 0.0010 | 0.0003 | 0.0002 | -0.0052 |
| MLP | 0.0899 | 0.0377 | 0.0283 | 0.0153 | 0.0070 | 0.0086 | 0.0038 | 0.0061 | 0.0002 | 0.0022 | -0.0006 | -0.0012 |
| CART | 0.0665 | 0.0450 | 0.0307 | 0.0145 | 0.0032 | 0.0016 | 0.0031 | 0.0000 | 0.0009 | 0.0000 | 0.0009 | 0.0000 |
| SVM | 0.0484 | 0.0632 | 0.0306 | 0.0121 | 0.0059 | -0.0030 | -0.0002 | 0.0000 | -0.0001 | 0.0002 | -0.0001 | 0.0003 |
| Stacking | 0.0482 | 0.0435 | 0.0307 | 0.0141 | 0.0046 | 0.0042 | 0.0018 | 0.0012 | 0.0003 | 0.0003 | 0.0001 | -0.0008 |

AI: artificial intelligence; CEA, carcinoembryonic antigen; LGBM, Light Gradient Boosting Decision; RF, Random Forest; GNB, Gaussian Naive Bayesian; KNN, K-Nearest Neighbor; MLP, Multilayer Perceptron; CART, Classification and Regression Trees; SVM, Support Vector Machine.
